# Ipsilateral hemiparesis caused by putaminal hemorrhage in a patient with horizontal gaze palsy with progressive scoliosis: a case report

**DOI:** 10.1186/s12883-015-0286-4

**Published:** 2015-03-10

**Authors:** Shuhei Yamada, Yoshiko Okita, Tomoko Shofuda, Ema Yoshioka, Masahiro Nonaka, Kosuke Mori, Shin Nakajima, Yonehiro Kanemura

**Affiliations:** Department of Neurosurgery, Osaka National Hospital, National Hospital Organization, 2-1-14 Hoenzaka, Chuo-ku, Osaka City, 540-0006 Japan; Division of Stem Cell Research, Institute for Clinical Research, Osaka National Hospital, National Hospital Organization, 2-1-14 Hoenzaka, Chuo-ku, Osaka City, 540-0006 Japan; Division of Regenerative Medicine, Institute for Clinical Research, Osaka National Hospital, National Hospital Organization, 2-1-14 Hoenzaka, Chuo-ku, Osaka City, 540-0006 Japan

**Keywords:** Putaminal hemorrhage, Ipsilateral hemiparesis, Horizontal gaze palsy with progressive scoliosis, Diffusion tensor imaging, *ROBO3* gene

## Abstract

**Background:**

Horizontal gaze palsy with progressive scoliosis (HGPPS) is an autosomal recessive disorder caused by mutations in the *ROBO3* gene, resulting in a critical absence of crossing fibers in the brainstem.

**Case presentation:**

We present a patient with ipsilateral hemiparesis caused by putaminal hemorrhage who had a history of horizontal gaze paralysis and scoliosis since childhood. Diffusion tensor imaging (DTI) tractography confirmed the presence of uncrossed corticospinal tracts. Sequence analysis of the entire *ROBO3* coding regions revealed a novel nonsense mutation.

**Conclusion:**

We report the first known HGPPS case with intracranial hemorrhage and *ROBO3* mutation showing an absence of major crossing pathways by DTI.

## Background

Horizontal gaze palsy with progressive scoliosis (HGPPS) is a rare congenital disorder with autosomal recessive inheritance that is associated with mutations in the *ROBO3* gene located at chromosome 11q23-25 [1]. HGPPS is characterized by the absence of conjugate horizontal eye movements, preservation of the vertical gaze and convergence, and progressive scoliosis during childhood and adolescence. The syndrome also includes a distinctive brain stem malformation and defective crossing of certain brain stem neuronal pathways. A few reports have used diffusion tensor imaging (DTI) to identify specific fiber tracts and their directionality in HGPPS and have shown the absence of major crossing pathways within the pons and midbrain [2-5].

There are a few reports of ipsilateral hemiplegia or hemiparesis following a supratentorial cerebral stroke [6-8]. Two case reports have described patients with ipsilateral hemiplegia or hemiparesis caused by intracranial hemorrhage [6,8], but mutations in the *ROBO3* gene were not examined in these cases. Only one report showed that HGPPS patients with *ROBO3* mutations displayed ischemic stroke symptoms on the ipsilateral side of the infarct [7].

We report the first known HGPPS case with intracerebral hemorrhage and mutation in the *ROBO3* gene showing the absence of major crossing pathways by DTI.

## Case presentation

**Figure 1:**
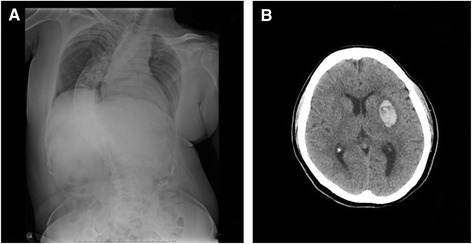
**Initial computed tomography (CT) and spinal radiography performed in a 55-year-old woman presenting with acute left hemiparesis. A)** Posterior-anterior spine radiography demonstrating scoliosis. **B)** Initial CT demonstrating putaminal hemorrhage.

**Figure 2:**
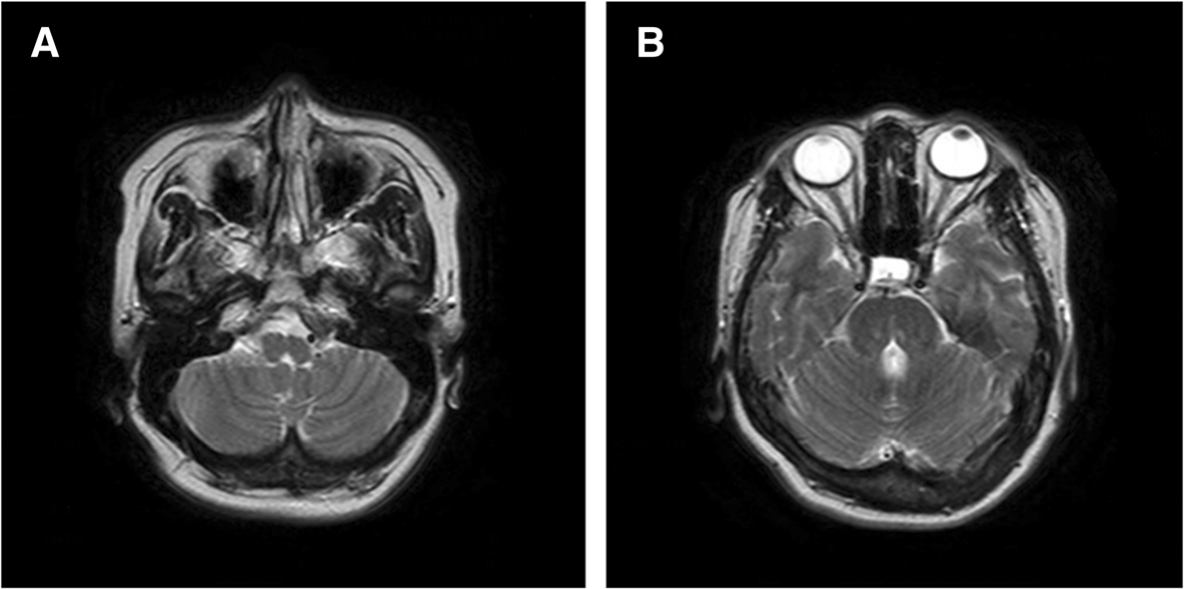
**Initial magnetic resonance imaging (MRI) demonstrating the hypoplastic pons and medulla in a 55-year-old woman with acute left hemiparesis.** The MRI shows a flattened “butterfly-like” medulla **(A)** and a split pons sign **(B)**, indicated by the deep midsagittal cleft extending ventrally from the fourth ventricular floor.

**Figure 3:**
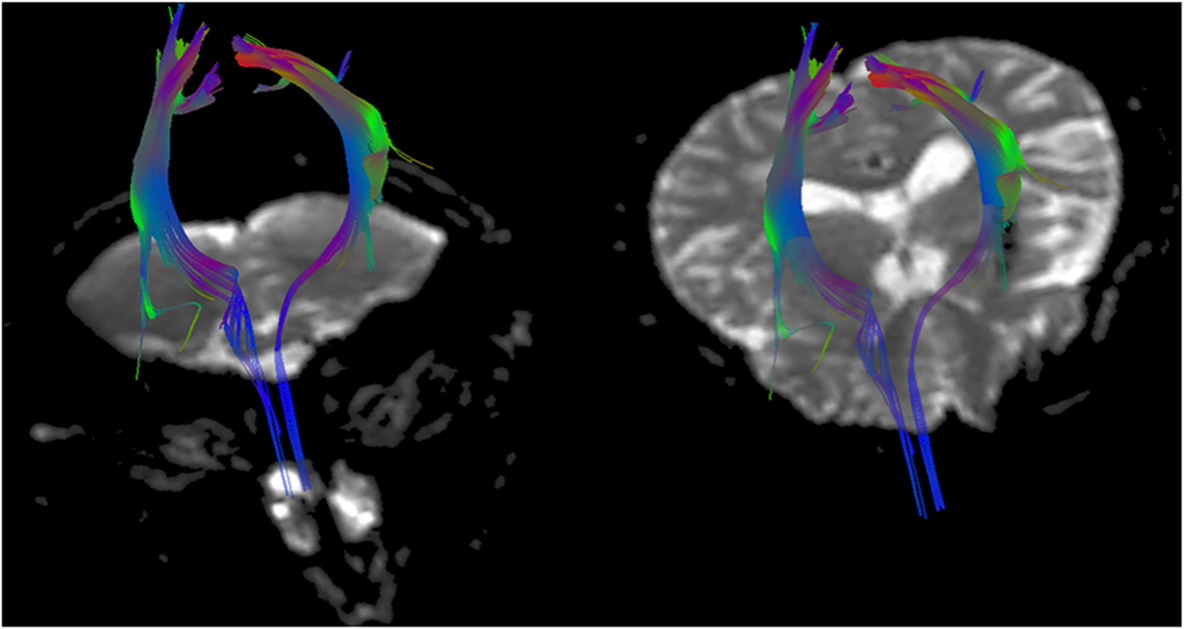
**Diffusion tensor imaging tractography showing uncrossed corticospinal tracts.**

A 55-year-old woman with a history of horizontal gaze paralysis and scoliosis (Figure 1A) since childhood was admitted to our hospital with a history of acute left hemiparesis. Computed tomography (CT) and magnetic resonance imaging (MRI) revealed left putaminal hemorrhage and brain stem hypoplasia (Figures 1B and 2). She is the first child of healthy parents who are second cousins, and her brother developed scoliosis in childhood. Diffusion tensor imaging (DTI) was performed to evaluate the corticospinal pathways. DTI tractography confirmed the presence of uncrossed corticospinal tracts (Figure 3).

### Genetic diagnosis

**Figure 4:**
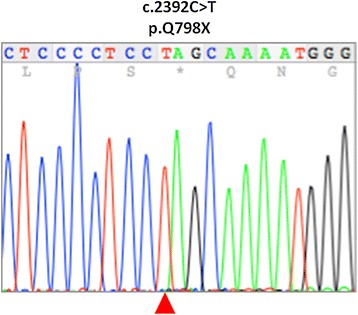
**Genetic analysis of the** ***ROBO3*** **gene.** Exon 15 of *ROBO3* shows a c.2392C > T (p.Q798X) nonsense mutation arrowhead).

Because she presented with clinical features suggestive of HGPPS, the entire coding region of *ROBO*3 was subjected to sequence analysis. Genetic testing was approved by the ethical committee of Osaka National Hospital (No.123) and was carried out in accordance with the principles of the Declaration of Helsinki, the Ethical Guidelines for Human Genome/Gene Analysis Research by the Ministry of Education, Culture, Science, and Technology, the Ministry of Health, Labor, and Welfare, and the Ministry of Economy, Trade, and Industry of Japan. After performing genetic counseling and obtaining written informed consent from the patient, genomic DNA was extracted from peripheral blood cells, and DNA sequencing was performed directly on the purified PCR products using a capillary DNA sequencer (3130xI Genetic Analyzer, Applied Biosystems). She carried a homozygous nonsense mutation c.2392C > T in exon 15 of *ROBO3* (Figure 4). This mutation has not been reported previously.

She was treated and showed significant clinical improvement, and she was subsequently discharged to home.

## Discussion

*ROBO3* aids in the regulation of hindbrain axonal midline crossing, helps direct cell migration, and specifies the lateral position of longitudinal pathways [1]. *ROBO3* mutations result in abnormal horizontal eye movement, progressive scoliosis, distinctive brain stem malformation, and defective crossing of select brain stem neuronal pathways. The *ROBO3* gene does not appear to have a region at high risk of mutation, and 32 different *ROBO3* mutations have been reported [1,7,9-15]. In our case, we found a novel homozygous nonsense mutation c.2392C > T in exon 15.

DTI is a unique tool able to map the white matter fiber tracts non-invasively and advances our understanding of abnormal brain anatomy [16,17]. In previous reports, DTI revealed the absence of major crossing pathways in the pons and midbrain in HGPPS [2-5]. Our case also showed uncrossed corticospinal tract on DTI.

Previously, only two reports described patients with ipsilateral hemiplegia or hemiparesis caused by intracranial hemorrhage [6,8]. Terakawa et al. described a patient with putaminal hemorrhage who had marked congenital scoliosis in the thoracolumbar spine and horizontal eye movement that was mildly restricted bilaterally [8]. Similarly, Hosokawa et al. described a patient with internal capsule and thalamic hemorrhage who had marked congenital scoliosis [6]. An MRI showed medulla hypoplasia in both cases. Mutations in the *ROBO3* gene were not examined in either case; however, these two cases suggested the clinical features of HGPPS. Only one report has identified *ROBO3* mutation in HGPPS patients presenting with ischemic stroke symptoms on the ipsilateral side of the infarct [7]. The present case is the first known report of hemorrhagic stroke with a confirmed *ROBO3* mutation and uncrossed corticospinal tracts resulting in ipsilateral putaminal hemorrhage and hemiparesis.

## Conclusion

We report the first HGPPS case with putaminal hemorrhage and *ROBO3* gene mutation showing an absence of major crossing pathways by DTI. Clinicians who encounter a patient with ipsilateral hemiparesis and intracerebral hemorrhage should suspect HGPPS and examine the patient for clinical and radiological features of HGPPS.

## Consent

We obtained written informed consent from the patient for publication of this case report and any accompanying images. A copy of the written consent is available for review by the Editor-in-Chief of this journal.
